# Calprotectin, a Promising Serological Biomarker for the Early Diagnosis of Superinfections with Multidrug-Resistant Bacteria in Patients with COVID-19

**DOI:** 10.3390/ijms25179294

**Published:** 2024-08-27

**Authors:** Dennis Keller, Patricia Mester, Ulrich Räth, Sabrina Krautbauer, Stephan Schmid, Verena Greifenberg, Martina Müller, Claudia Kunst, Christa Buechler, Vlad Pavel

**Affiliations:** 1 Department of Internal Medicine I, Gastroenterology, Hepatology, Endocrinology, Rheumatology, and Infectious Diseases, University Hospital Regensburg, 93053 Regensburg, Germany; dennis.keller@stud.uni-regensburg.de (D.K.); patricia.mester@klinik.uni-regensburg.de (P.M.); ulrich.raeth@stud.uni-regensburg.de (U.R.); stephan.schmid@klinik.uni-regensburg.de (S.S.); martina.mueller-schilling@klinik.uni-regensburg.de (M.M.); claudia.kunst@klinik.uni-regensburg.de (C.K.); vlad.pavel@klinik.uni-regensburg.de (V.P.); 2 Institute of Clinical Chemistry and Laboratory Medicine, University Hospital Regensburg, 93053 Regensburg, Germany; sabrina.krautbauer@klinik.uni-regensburg.de; 3 Institute of Clinical Microbiology and Hygiene, University Hospital Regensburg, 93053 Regensburg, Germany; verena.greifenberg@klinik.uni-regensburg.de

**Keywords:** calprotectin, biomarker, critical care, bloodstream infections, COVID-19

## Abstract

Bacterial and fungal superinfections are common in COVID-19, and early diagnosis can enable timely intervention. Serum calprotectin levels increase with bacterial, fungal, and viral infections. This study evaluated serum calprotectin as a diagnostic and prognostic tool for microbial superinfections in COVID-19. Serum samples from adult patients with moderate and severe COVID-19 were collected during hospitalization from 2020 to 2024. Calprotectin levels were measured using an enzyme-linked immunosorbent assay in 63 patients with moderate COVID-19, 60 patients with severe COVID-19, and 34 healthy individuals. Calprotectin serum levels were elevated in patients with moderate COVID-19 compared with controls, and these levels were further increased in the severe cases. Patients with severe COVID-19 and vancomycin-resistant enterococci (VRE) bacteremia had elevated calprotectin levels, but their C-reactive protein and procalcitonin levels were not increased. Fungal superinfections and herpes simplex virus reactivation did not change the calprotectin levels. A calprotectin concentration of 31.29 µg/mL can be used to diagnose VRE bloodstream infection with 60% sensitivity and 96% specificity. These data suggest that serum calprotectin may be a promising biomarker for the early detection of VRE bloodstream infections in patients with COVID-19.

## 1. Introduction

SARS-CoV-2 was discovered in Wuhan, China, in 2019 and spread rapidly, causing a global COVID-19 pandemic [1,2]. Evidence suggests that a dysregulated immune response causes a cytokine storm in patients with COVID-19, worsening the disease [3,4]. Additionally, SARS-CoV-2 infection damages tissues and induces the recruitment and activation of immune cells, particularly monocytes and neutrophils [5,6,7].

High neutrophil counts are linked to severe COVID-19 and trigger a significant release of calprotectin (S100A8/S100A9) [5,6,7,8,9]. Recent meta-analyses and studies report elevated serum calprotectin levels in patients with COVID-19, especially those in intensive care units [6,10,11,12,13,14,15]. Calprotectin is also associated with the serious adverse effects of SARS-CoV-2 vaccination, and a marked increase in calprotectin has been described in patients with vaccine-induced immune thrombotic thrombocytopenia [16].

The SARS-CoV-2 spike protein induces calprotectin production in neutrophils via the activation of Toll-like receptor 4 (TLR4) [17]. As an endogenous TLR4 ligand, calprotectin contributes to excessive inflammation [18]. 

Calprotectin is a highly abundant cytosolic protein of neutrophil granulocytes, accounting for 40–50% of the total protein content, and it is released during neutrophil activation and turnover. Thus, it is a crucial marker of neutrophil-induced inflammation [19]. Calprotectin, a heterodimer of S100A8 and S100A9 (also known as calgranulin A and B) [9], has antimicrobial functions due to its ability to bind divalent cations, such as copper, calcium, manganese, and zinc. Its antimicrobial effects that do not involve metal chelation are poorly understood [20,21]. Calprotectin induces the production of interferon-gamma, a cytokine with antiviral activities, in natural killer cells [22,23]; this suggests that the upregulation of calprotectin may be a protective mechanism. However, endothelial dysfunction in patients with COVID-19, which contributes to oxidative stress, inflammation, and thrombosis, is exaggerated by calprotectin, and it has been shown to disturb mitochondrial function and induce PANoptosis in endothelial cells [24,25]. Calprotectin can also activate the nuclear factor kappa B pathway in the lungs, promoting the inflammatory polarization of macrophages, epithelial cell apoptosis, and lung injury [26,27]. Autoantibodies to calprotectin are common in patients with COVID-19 and are associated with better recovery eight months after infection [28]. 

Serum calprotectin is induced by inflammatory stimuli and is an early marker of bacterial and viral infections [29,30]. Blood calprotectin is a good biomarker for bacterial infection, sepsis prediction, and mortality [31]. Calprotectin is best described as a fecal biomarker of intestinal inflammation, and the quantity of fecal calprotectin is strongly elevated in patients with severe COVID-19 [32]. 

Bacterial superinfection within the first 48 h of ICU admission occurs in 23% to 28% of patients with COVID-19 [33,34]. A meta-analysis reported a 20% prevalence of superinfections in hospitalized and non-hospitalized patients with COVID-19, with fungal superinfections in 8% and viral superinfections in 4% [35]. Herpes simplex virus (HSV) reactivation is also associated with COVID-19 [36]. An Italian study found that nearly 30% of invasively ventilated patients with COVID-19 with worsening respiratory function experienced HSV-1 reactivation [37].

Superinfections are more common in severe COVID-19 cases, worsening the disease and increasing the risk of death [35,38,39]. Therefore, higher serum calprotectin levels in severe cases may partly result from fungal and bacterial superinfections.

There is a limited understanding of how infection with SARS-CoV-2 impairs the function of the innate immune system against bacterial, fungal, and viral superinfections. Changes in thrombopoiesis, granulopoiesis, and erythropoiesis indicate serious perturbations of all blood cells in COVID-19 [40]. Neutrophil function in patients with COVID-19 is impaired, reducing reactive oxygen species production and myeloperoxidase activity when challenged with bacteria [41,42], indicating that superinfection may not be related to the further rise in calprotectin. 

Calprotectin levels in cases of non-COVID-19 acute respiratory infections are significantly higher in patients with infections such as mycoplasma pneumonia, bacterial pneumonia, and streptococcal tonsillitis compared with patients with viral infections. Rapidly measuring calprotectin levels can aid in managing respiratory infections, enabling more accurate diagnosis and selective antibiotic use [30]. Plasma calprotectin is also an early marker of bacterial infections in severely ill patients [43]. Another study described higher plasma calprotectin levels in patients with bacterial infections, as compared with non-infected patients. In this cohort, the difference between non-infected controls and patients with viral infections was not significant [44]. *Candida albicans* blood infection activates neutrophils, releasing calprotectin, indicating that serum calprotectin may be induced in fungal infections [45]. 

Blood calprotectin is higher in patients with mild COVID-19 disease than in controls and is significantly increased in severe cases, requiring hospitalization [42,46,47]. The increase in calprotectin is independent of factors such as older age, comorbidities, and bacterial infections [42]. Increased plasma calprotectin during severe COVID-19 is related to an unfavorable outcome and mortality [48,49]. However, the association of superinfections in COVID-19 with circulating calprotectin levels has not been studied in much detail [18]. 

Superinfections in patients with SARS-CoV-2 impact treatment decisions. However, proving infection quickly and accurately remains challenging [50]. We aimed to evaluate serum calprotectin levels via enzyme-linked immunosorbent assay in patients with moderate and severe COVID-19 to clarify whether calprotectin levels are further increased by bacterial and/or fungal superinfections or HSV reactivation. 

## 2. Results

### 2.1. Study Cohorts

This study included 60 patients with severe COVID-19, 63 with moderate COVID-19, and 34 healthy controls. The criteria for systemic inflammatory response syndrome (SIRS) were fulfilled by patients with moderate COVID-19 [51,52]. This group is considered to be clinically equivalent to moderate COVID-19 [53]. The patients were closely monitored during their hospitalization but did not require admission to the intensive care unit (ICU). Patients with septic shock and acute respiratory distress syndrome were admitted to the ICU. These patients correspond to severe illness according to the National Institutes of Health classification [52,53,54,55]. Blood from patients with severe COVID-19 was sampled between 16 April 2020 and 14 June 2021. In Germany, vaccination against SARS-CoV-2 began on 26 December 2020, and almost all of our severe patients were not vaccinated. The patients were treated according to the approved guidelines of the European Medicines Agency and the German Federal Joint Committee. At that time, the drugs approved for COVID-19 treatment were remdesivir and dexamethasone. All patients received unfractionated heparin or low-molecular-weight heparin for thrombosis prophylaxis. 

The 34 healthy controls (15 males and 19 females) had a similar sex distribution and ages compared with the patients with moderate and severe COVID-19 (Table 1). 

In patients with severe COVID-19, ferritin, C-reactive protein (CRP), procalcitonin, and lactate dehydrogenase (LDH) levels were higher than those in patients with moderate disease (Table 1). The two cohorts were of similar ages and had a comparable gender distribution. The body mass index (BMI) of patients with severe disease was high. Alkaline phosphatase (AP) and interleukin (IL)-6 levels were almost identical between the two COVID-19 groups (Table 1). The neutrophil, basophil, monocyte, and immature granulocyte counts of patients with severe COVID-19 were increased, whereas eosinophils and lymphocytes did not differ between the groups. Viral load was similar between the patient cohorts, and antibody titers were significantly elevated in severe cases (Table 1). 

### 2.2. Serum Calprotectin Levels of Healthy Controls and Patients 

**Figure 1:**
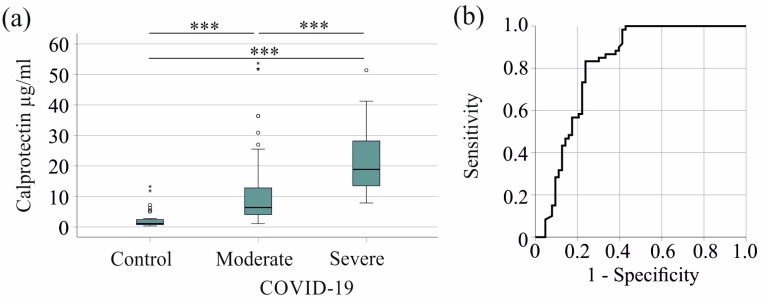
Serum calprotectin increases in patients with COVID-19. (**a**) Serum calprotectin levels in control subjects, patients with moderate COVID-19, and patients with severe COVID-19; (**b**) receiver operating characteristic curve distinguishing between moderate and severe COVID-19 patients. *** *p* < 0.001.

Serum calprotectin levels were elevated in patients with severe COVID-19 compared with controls and patients with moderate COVID-19. The median serum calprotectin levels were 1.11 (0.32–13.26) µg/mL for the controls, 6.34 (1.09–53.67) µg/mL for patients with moderate COVID-19, and 18.83 (7.84–51.36) µg/mL for severe cases (Figure 1a).

Serum calprotectin showed an area under the receiver operating characteristic curve (AUROC) of 0.812 for the prediction of severe COVID-19 (*p* < 0.001) (Figure 1b). A 12.90 µg/mL serum calprotectin value had a sensitivity of 83% and a specificity of 76% for discriminating moderate and severe disease. 

### 2.3. Serum Calprotectin Levels in Relation to Age, Sex, and Body Mass Index 

In the moderate group, serum calprotectin levels were not related to age (r = −0.012, *p* = 0.928), BMI (r = −0.033, *p* = 0.859), or sex (*p* = 0.558). There was no sex disparity in severe COVID-19 (*p* = 0.073). Serum calprotectin did not correlate with age (r = 0.136, *p* = 0.305) or BMI (r = 0.035, *p* = 0.796) in this group. 

### 2.4. Serum Calprotectin Levels in Relation to Viral Load and Antibody Titer

In moderate (r = 0.234, *p* = 0.082) and severe (r = 0.028, *p* = 0.853) COVID-19, serum calprotectin was not related to viral load and did not correlate with antibody titers (r = 0.176, *p* = 0.627 and r = 0.119, *p* = 0.415, respectively).

### 2.5. Calprotectin in Relation to Dialysis and Vasopressor Therapy in Severe COVID-19

In the moderate COVID-19 group, no patient received vasopressor therapy or required ventilation. The six patients requiring dialysis had similar serum calprotectin to those without dialysis requirements (Table 2). This also applied to the 7 patients with severe COVID-19 on dialysis (Table 2) and the 41 patients requiring vasopressor therapy (Table 2). As only one patient with severe COVID-19 disease did not need invasive ventilation, we could not perform a statistical analysis comparing patients with and without ventilation.

### 2.6. Correlation of Serum Calprotectin with Inflammatory Markers and White Blood Cell Count

Serum calprotectin positively correlated with CRP in the moderate and severe groups and procalcitonin in the latter cohort. In contrast, calprotectin levels were unrelated to IL-6 and ferritin in moderate or severe COVID-19 (Table 3).

Notably, serum calprotectin did not correlate with white blood cell counts in severe COVID-19. However, in the moderate group, neutrophils (r = 0.620, *p* < 0.001), basophils (r = 0.258, *p* = 0.041), monocytes (r = 0.340, *p* = 0.006), lymphocytes (r = 0.268, *p* = 0.034), and immature granulocyte (r = 0.593, *p* < 0.001) positively correlated with serum calprotectin levels. 

### 2.7. Relationship of Serum Calprotectin to Bacterial and Fungal Superinfections and HSV Reactivation

Patients with severe COVID-19 had significantly more superinfections with fungi (*p* < 0.001), vancomycin-resistant enterococci (VRE) (*p* = 0.001), or bacteria in general (*p* < 0.001). HSV reactivation was also significantly increased (*p* < 0.001) in this group.

In patients with moderate COVID-19, bacterial superinfection did not alter serum calprotectin levels in six patients (*p* = 0.499). Two patients had fungal infections and none of the patients had HSV reactivation. 

**Figure 2:**
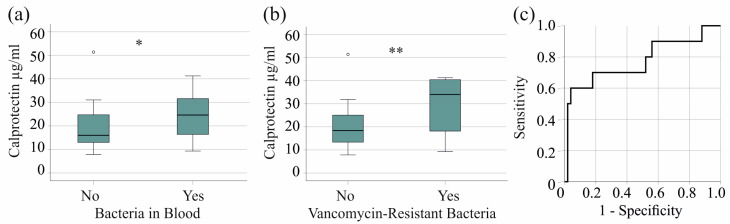
Serum calprotectin levels in patients with severe COVID-19 and bacterial superinfections. (**a**) Serum calprotectin levels of patients without (No) and with (Yes) bacteriemia; (**b**) serum calprotectin levels of patients without (No) and with (Yes) vancomycin-resistant bacteria in their blood; (**c**) ROC curve to distinguish between patients with and without vancomycin-resistant bacteria. * *p* < 0.05, ** *p* < 0.01.

In the severe COVID-19 group, 27 patients had bacterial bloodstream infections, and the serum calprotectin of infected patients was significantly increased (*p* = 0.023) (Figure 2a). The 10 patients with VRE in their blood had higher serum calprotectin levels (*p* = 0.007) (Figure 2b). Excluding VRE did not reveal elevated serum calprotectin levels in patients with bacterial bloodstream infections (*p* = 0.250), indicating that this effect is specific to VRE. The AUROC of serum calprotectin to discriminate patients with and without VRE bloodstream infections was 0.771 (*p* = 0.007). A calprotectin concentration of 31.29 µg/mL showed 60% sensitivity and 96% specificity for diagnosing VRE in patients with severe COVID-19 (Figure 2c).

**Figure 3:**
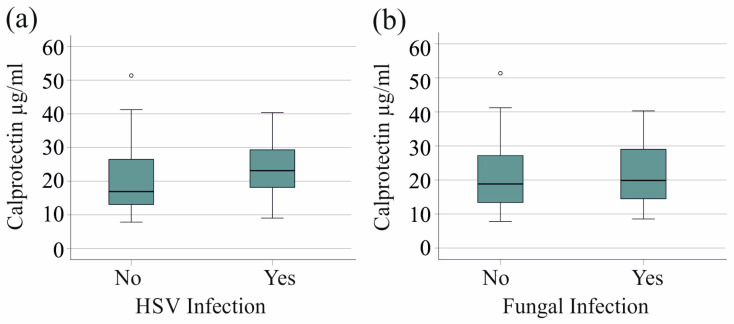
Serum calprotectin levels of patients with severe COVID-19 and herpes simplex virus (HSV) reactivation or fungal superinfections. (**a**) Serum calprotectin levels of patients with (Yes) and without (No) HSV reactivation; (**b**) serum calprotectin levels of patients with (Yes) and without (No) fungal infection.

We found no association between HSV reactivation in 20 patients with severe COVID-19 and serum calprotectin levels (*p* = 0.079). Furthermore, the 21 patients with fungal superinfections exhibited serum calprotectin levels that were comparable to those observed in patients without fungal infections (*p* = 0.718) (Figure 3a,b).

CRP and procalcitonin levels in patients with bacterial, VRE, and fungal superinfections, as well as those with HSV reactivation, were similar to those in patients without these infections. Regarding immune cells, only the number of immature granulocytes tended to be increased in VRE-infected patients with severe COVID-19 (*p* = 0.061).

### 2.8. Serum Calprotectin and Survival

**Figure 4:**
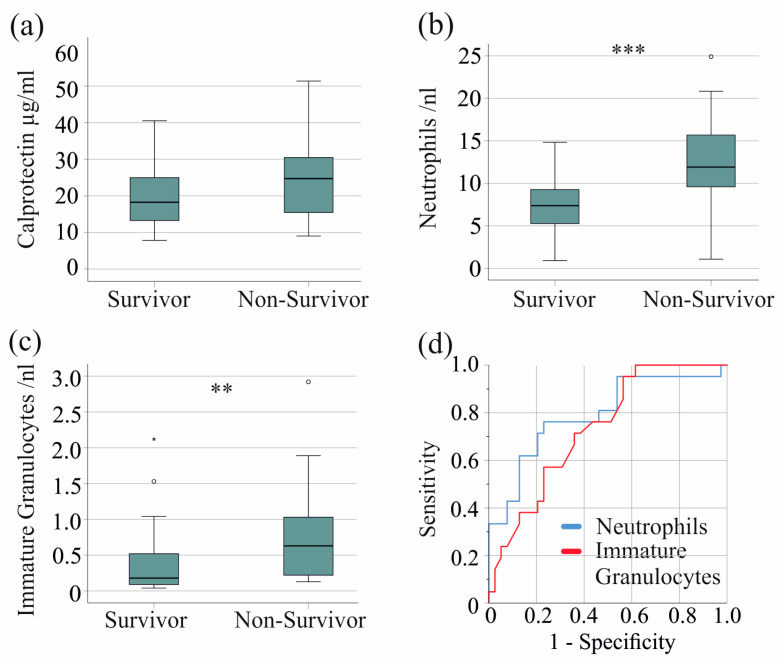
Serum calprotectin, neutrophil, and immature granulocyte count and survival of patients with severe COVID-19. (**a**) Serum calprotectin levels of surviving and non-surviving patients; (**b**) neutrophils of surviving and non-surviving patients; (**c**) immature granulocytes of surviving and non-surviving patients; (**d**) ROC curve to distinguish between survivors and non-survivors. ** *p* < 0.01, *** *p* < 0.001.

In the severe COVID-19 patient group, 21 patients died. Serum calprotectin levels were comparable between non-survivors and survivors with severe COVID-19 (*p* = 0.079) (Figure 4a). Of clinical relevance, in the entire cohort, including patients with moderate disease, non-survivors had significantly higher serum calprotectin levels (*p* = 0.001).

Notably, neutrophil (*p* < 0.001) and immature granulocyte counts (*p* = 0.005) were increased in non-survivors, whereas monocyte, basophil, eosinophil, and lymphocyte counts were not. The AUROC for the neutrophil count was 0.792 (Figure 4b,d), with a threshold of 9.5 neutrophils/nL, discriminating survivors from non-survivors with 76% sensitivity and 77% specificity. The AUROC for the immature granulocyte count was 0.732 (Figure 4c,d), with a threshold of 0.15 immature granulocytes/nL discriminating survivors from non-survivors with 95% sensitivity and 44% specificity.

## 3. Discussion

This study identified serum calprotectin as a valuable tool for the early diagnosis of bloodstream superinfections with VRE in patients with severe COVID-19. Notably, serum calprotectin was not elevated in COVID-19 patients with other bacterial superinfections, such as *Escherichia coli*, fungal superinfections, or HSV reactivation, indicating its specificity for VRE.

There is compelling evidence that calprotectin is induced in SARS-CoV-2 infection and is related to disease severity [6,14,56,57]. In our cohort, serum calprotectin levels were increased in patients with moderate COVID-19 disease compared with controls and were further elevated in severe cases. This suggests that serum calprotectin could serve as a valuable biomarker for disease monitoring.

Calprotectin is a well-described biomarker for bacterial and viral infections that can serve as a diagnostic tool to facilitate antibiotic treatment decisions [29,58]. 

A retrospective cohort study showed that nearly 5% of hospitalized patients with COVID-19 acquired bacterial superinfections [38]. A meta-analysis reported a bacterial superinfection rate of 20%, a fungal superinfection rate of 8%, and a viral superinfection rate of 4% [35]. 

Fungal and bacterial superinfections did not further elevate serum calprotectin in our patients with severe COVID-19. Similarly, HSV reactivation did not increase serum calprotectin levels. Therefore, the higher serum calprotectin observed in bloodstream superinfections with VRE appears to be VRE-specific. Interestingly, the number of neutrophils, the main cellular source of calprotectin [9], did not differ between patients with and without VRE in severe COVID-19. This suggests that VRE triggers calprotectin release without increasing neutrophil numbers. To the best of our knowledge, these observations have not been previously described. 

Notably, the CRP and procalcitonin levels observed in patients with severe COVID-19 infections and HSV reactivation, or with fungal, bacterial, or VRE superinfections, were comparable to those observed in patients without these complications. Therefore, according to our data, CRP and procalcitonin cannot be used as early biomarkers for the detection of superinfections and HSV reactivation. Another study suggests that procalcitonin and CRP are elevated in COVID-19 patients with secondary bacterial infections. This analysis included patients with severe and moderate COVID-19, with the latter having lower CRP and procalcitonin levels and fewer superinfections [59]. In 48 critically ill COVID-19 patients, procalcitonin did not differ significantly between patients with and without bacterial superinfection, whereas CRP was lower in the latter group [60]. The corresponding *p*-value was 0.034, indicating that CRP is not an excellent marker. Similarly, critical COVID-19 patients with infections had modestly higher CRP levels (*p* = 0.046) than non-infected patients [61]. Moreover, CRP levels were not significantly increased in severe COVID-19 patients with HSV reactivation [62,63], whereas no data were found on procalcitonin levels. 

Since the initial isolation of VRE in England in 1988, this organism has spread at an alarming rate, becoming a predominant cause of nosocomial infections globally [64]. Vancomycin has traditionally been used to treat Gram-positive bacterial infections [65]. However, with the rising prevalence of antibiotic use, enterococci have evolved to possess genes that confer resistance to a range of antibiotics, including vancomycin. This represents a considerable risk to patient safety [64,65,66]. 

Various assays, such as PCR, electrochemical DNA biosensors, and immunoassays have been described for the detection of VRE [66]. These methods can detect VRE within 1 to 3 days, and all have drawbacks, such as high intra-assay variations and RNA instability. However, fecal calprotectin measurement is a routine analysis [67,68] and can be easily established for serum calprotectin levels.

Calprotectin is a prognostic marker for COVID-19 mortality. The study cohort comprised 195 adult patients, of whom 156 were hospitalized in the infectious disease unit and 39 in the ICU [6]. Another study reported the association of calprotectin with survival, including patients with both severe and non-severe COVID-19 [13]. The higher calprotectin levels observed in non-survivors are thus associated with disease severity rather than survival within a cohort of similarly ill patients, such as ours. Including both moderate and severe patients in our analysis also revealed an increase in calprotectin levels, consistent with previous studies [6,13]. 

Cardiero et al. identified neutrophil count as a survival marker [6]. Similarly, our study found increased neutrophil counts in non-survivors with severe COVID-19. Additionally, the number of immature granulocytes was elevated in non-survivors. In our severe COVID-19 cohort, a neutrophil count of 9.5 cells/nL discriminated survivors from non-survivors with 76% sensitivity and 77% specificity, while 0.15 immature granulocytes/nL showed 95% sensitivity and 44% specificity. Previous studies have also described the association between immature granulocyte count and survival in COVID-19, and our results are consistent with these findings [8,69]. 

Sex, age, and BMI did not correlate with serum calprotectin levels in moderate and severe COVID-19 cases. Similar results have been seen in critically ill patients [70], showing that these factors do not affect the link between serum calprotectin levels and how severe the patient’s condition is.

This study has limitations. Blood was not always collected early after hospital admission, and the vaccination status of some patients with moderate disease recruited in 2024 was not documented. This was a retrospective, monocenter study, which is a further limitation. In addition, the number of VRE patients was small, and our observation needs confirmation in larger cohorts. 

Future studies to validate serum calprotectin as a biomarker for the early detection of VRE infection should be prospective and multicentered. A standardized method of measuring serum calprotectin should be used to compare results between different centers. 

## 4. Materials and Methods

### 4.1. Study Cohort

Blood samples from 194 patients aged 18 years or older were collected at the University Hospital of Regensburg between 16 April 2020 and 12 January 2024. Blood was collected from all patients willing to participate in the study, and samples from 123 patients were randomly selected for this analysis to include a similar number of patients with moderate and severe disease. There were no inclusion or exclusion criteria other than age. Blood samples from healthy controls aged 18 years or older were collected from students and hospital staff.

### 4.2. Measurement of Serum Calprotectin

Serum calprotectin was determined using an enzyme-linked immunosorbent assay (ELISA). The IDK^®^ Calprotectin ELISA kit was used (Catalog Number: K 6935, Immundiagnostik AG, Bensheim, Germany). Each serum sample was tested twice, and the mean value was used for further calculations. To measure serum calprotectin, a dilution of 1:100 was prepared. 

### 4.3. Microbiological Tests

The diagnosis of bacterial infections was performed at the Institute of Clinical Microbiology and Hygiene, University Hospital Regensburg [71]. Enterococci and fungi were identified with matrix-assisted laser desorption/ionization time-of-flight (MALDI-TOF) mass spectrometry (Bruker Microflex LT; Bruker, Hamburg, Germany). Antimicrobial susceptibilities were determined via minimum inhibitory concentration measurement according to the European Committee on Antimicrobial Susceptibility Testing or with automated systems (Phoenix; BD, Franklin Lakes, NJ, USA). In the case of enterococci, vancomycin resistance was confirmed by detecting the van A and/or van B genes via PCR. Table S1 shows the antimicrobial susceptibility of the VRE-pathogens. Herpes simplex virus reactivation was detected using PCR.

### 4.4. Statistical Analysis

The data presented in the figures are illustrated as boxplots, showing the minimum, maximum, median, and first and third quartiles. Outliers are indicated with circles (calprotectin levels >1.5× the interquartile range from either quartile) and asterisks (calprotectin levels >3.0× the interquartile range from either quartile). The data in the table display the median, minimum, and maximum values. Statistical analyses were conducted using the Chi-square test, receiver operating characteristics curve, the Mann–Whitney U test, the Kruskal–Wallis test, and Spearman’s correlation in IBM SPSS Statistics 26.0. A *p*-value of <0.05 was considered to indicate a significant difference.

## 5. Conclusions

In conclusion, this study corroborates the notion that serum calprotectin is a valuable biomarker for disease monitoring in patients with COVID-19. It also provides initial evidence showing that serum calprotectin can serve as an early biomarker for diagnosing bloodstream superinfections with VRE in patients with severe forms of the disease. Serum calprotectin levels were not elevated in patients with superinfections from other bacteria and fungi or HSV reactivation, indicating their specificity for VRE. Further research is required to investigate whether serum calprotectin could be a valuable biomarker for multidrug-resistant bacteria in general.

The discovery of new biomarkers will facilitate more rational antibiotic use, which, in turn, will reduce bacterial resistance, hospitalization time, and mortality among patients infected with multidrug-resistant bacteria.

## Figures and Tables

**Table 1:** Characteristics of patients with COVID-19, as well as the gender and age of the controls. Superscript numbers refer to the number of patients for whom these measures were known (alkaline phosphatase: AP; arbitrary unit: AU; body mass index: BMI; interleukin: IL; lactate dehydrogenase: LDH).

Parameter	Moderate COVID-19	Severe COVID-19	Controls	*p*-Value
Males/Females (%)	34/29 (54/46)	42/18 (70/30)	15/19 (44/56)	0.116
Age (years)	60 (22–83)	57 (31–83)	56 (50–81)	0.492
BMI (kg/m^2^)	26.3 (18.4–42.6) ^32^	29.4 (19.2–66.7) ^56^	Not determined	0.007
C-reactive protein mg/L	26 (0–222)	74 (1–367)	Not determined	<0.001
Procalcitonin ng/ml	0.09 (0–24.90)	0.24 (0.06–25.00)	Not determined	<0.001
LDH U/L	224 (127–929) ^39^	378 (162–1534)	Not determined	<0.001
AP U/L	96 (38–372) ^29^	99 (37–743)	Not determined	0.943
Ferritin ng/mL	573 (32–4826) ^45^	1088 (77–21976) ^60^	Not determined	<0.001
IL-6 pg/mL	19 (4–265) ^37^	36 (3–1175)	Not determined	0.097
Neutrophils n/nL	4.05 (0.13–23.10)	8.18 (0.90–24.91)	Not determined	<0.001
Basophils n/nL	0.03 (0–0.21)	0.05 (0.01–0.17)	Not determined	0.001
Eosinophils n/nL	0.08 (0–1.19)	0.04 (0–1.07)	Not determined	0.223
Monocytes n/nL	0.57 (0.07–2.52)	0.71 (0.03–2.21)	Not determined	0.042
Lymphocytes n/nL	1.11 (0.09–57.83)	1.20 (0–75.95)	Not determined	0.846
Immature Granulocytes n/nL	0.03 (0–1.38)	0.25 (0.04–2.92)	Not determined	<0.001
Viral Load	8600 (48–19 × 10^6^) ^57^	14000 (95–52 × 10^7^) ^49^	Not determined	0.090
Antibody AU/mL	101 (14–1487) ^10^	661 (17–1939) ^50^	Not determined	0.037

**Table 2:** Serum calprotectin levels (µg/mL) did not change with dialysis or vasopressor therapy.

Intervention/Drug	No	Yes
Moderate		
Dialysis (6 patients)	6.34 (1.09–53.67)	12.19 (3.94–51.68)
Severe		
Dialysis (7 patients)	18.55 (7.84–51.36)	24.70 (9.02–40.29)
Catecholamine (41 patients)	17.26 (8.16–40.50)	21.36 (7.84–51.36)

**Table 3:** Spearman correlation coefficients for the associations of serum calprotectin with inflammatory markers. ** *p* < 0.01, *** *p* < 0.001.

C-Reactive Protein		Procalcitonin		IL-6		Ferritin	
Moderate	Severe	Moderate	Severe	Moderate	Severe	Moderate	Severe
0.350 **	0.354 **	0.215	0.437 ***	0.007	0.236	0.226	0.181

## Data Availability

The authors confirm that the data supporting the results of this study are available within the article. The raw data are available from the corresponding author upon reasonable request.

## Supplementary Materials

The following supporting information can be downloaded at https://www.mdpi.com/article/10.3390/ijms25179294/s1.

## References

[1] Borges do Nascimento I.J., Cacic N., Abdulazeem H.M., von Groote T.C., Jayarajah U., Weerasekara I., Esfahani M.A., Civile V.T., Marusic A., Jeroncic A. (2020). Novel Coronavirus Infection (COVID-19) in Humans: A Scoping Review and Meta-Analysis. J. Clin. Med..

[2] Huang C., Wang Y., Li X., Ren L., Zhao J., Hu Y., Zhang L., Fan G., Xu J., Gu X. (2020). Clinical features of patients infected with 2019 novel coronavirus in Wuhan, China. Lancet.

[3] Bhaskar S., Sinha A., Banach M., Mittoo S., Weissert R., Kass J.S., Rajagopal S., Pai A.R., Kutty S. (2020). Cytokine Storm in COVID-19-Immunopathological Mechanisms, Clinical Considerations, and Therapeutic Approaches: The REPROGRAM Consortium Position Paper. Front. Immunol..

[4] Georgieva E., Ananiev J., Yovchev Y., Arabadzhiev G., Abrashev H., Abrasheva D., Atanasov V., Kostandieva R., Mitev M., Petkova-Parlapanska K. (2023). COVID-19 Complications: Oxidative Stress, Inflammation, and Mitochondrial and Endothelial Dysfunction. Int. J. Mol. Sci..

[5] Bean J., Kuri-Cervantes L., Pennella M., Betts M.R., Meyer N.J., Hassan W.M. (2023). Multivariate indicators of disease severity in COVID-19. Sci. Rep..

[6] Cardiero G., Palma D., Vano M., Anastasio C., Pinchera B., Ferrandino M., Gianfico C., Gentile L., Savoia M., Gentile I. (2022). Calprotectin Levels and Neutrophil Count Are Prognostic Markers of Mortality in COVID-19 Patients. Diagnostics.

[7] McKenna E., Wubben R., Isaza-Correa J.M., Melo A.M., Mhaonaigh A.U., Conlon N., O’Donnell J.S., Ni Cheallaigh C., Hurley T., Stevenson N.J. (2022). Neutrophils in COVID-19: Not Innocent Bystanders. Front. Immunol..

[8] Meizlish M.L., Pine A.B., Bishai J.D., Goshua G., Nadelmann E.R., Simonov M., Chang C.H., Zhang H., Shallow M., Bahel P. (2021). A neutrophil activation signature predicts critical illness and mortality in COVID-19. Blood Adv..

[9] Yui S., Nakatani Y., Mikami M. (2003). Calprotectin (S100A8/S100A9), an inflammatory protein complex from neutrophils with a broad apoptosis-inducing activity. Biol. Pharm. Bull..

[10] Chapuis N., Ibrahimi N., Belmondo T., Goulvestre C., Berger A.E., Mariaggi A.A., Andrieu M., Chenevier-Gobeaux C., Bayle A., Campos L. (2022). Dynamics of circulating calprotectin accurately predict the outcome of moderate COVID-19 patients. EBioMedicine.

[11] Gatselis N.K., Lyberopoulou A., Lygoura V., Giannoulis G., Samakidou A., Vaiou A., Antoniou K., Triantafyllou K., Stefos A., Georgiadou S. (2023). Calprotectin serum levels on admission and during follow-up predict severity and outcome of patients with COVID-19: A prospective study. Eur. J. Intern. Med..

[12] Infantino M., Manfredi M., Alessio M.G., Previtali G., Grossi V., Benucci M., Faraone A., Fortini A., Grifoni E., Masotti L. (2022). Clinical utility of circulating calprotectin to assist prediction and monitoring of COVID-19 severity: An Italian study. J. Med. Virol..

[13] Kumar T., Bhushan D., Kumar S., Jha K., Verma P., Ganguly A., Kumar Y., Zabihullah M. (2022). Role of cystatin C and calprotectin as potential early prognostic biomarkers in COVID-19 patients admitted to a dedicated COVID care facility. J. Fam. Med. Prim. Care.

[14] Mao Q., Wang C., Wen W., Zhou M., Tang J., Chen C., Cheng Y., Wu Q., Zhang X., Feng Z. (2022). A meta-analysis of the association between calprotectin and the severity of COVID-19. J. Infect..

[15] Norman G.L., Navaz S.A., Kanthi Y., Albesa R., Mahler M., Knight J.S., Zuo Y. (2022). Circulating Calprotectin as a Predictive and Severity Biomarker in Patients with COVID-19. Diagnostics.

[16] Hetland G., Fagerhol M.K., Wiedmann M.K.H., Soraas A.V.L., Mirlashari M.R., Nissen-Meyer L.S.H., Istre M.S., Holme P.A., Schultz N.H. (2022). Elevated NETs and Calprotectin Levels after ChAdOx1 nCoV-19 Vaccination Correlate with the Severity of Side Effects. Vaccines.

[17] Loh J.T., Teo J.K.H., Lam K.P. (2022). Dok3 restrains neutrophil production of calprotectin during TLR4 sensing of SARS-CoV-2 spike protein. Front. Immunol..

[18] Mellett L., Khader S.A. (2022). S100A8/A9 in COVID-19 pathogenesis: Impact on clinical outcomes. Cytokine Growth Factor Rev..

[19] Pruenster M., Vogl T., Roth J., Sperandio M. (2016). S100A8/A9: From basic science to clinical application. Pharmacol. Ther..

[20] Besold A.N., Culbertson E.M., Nam L., Hobbs R.P., Boyko A., Maxwell C.N., Chazin W.J., Marques A.R., Culotta V.C. (2018). Antimicrobial action of calprotectin that does not involve metal withholding. Metallomics.

[21] Besold A.N., Gilston B.A., Radin J.N., Ramsoomair C., Culbertson E.M., Li C.X., Cormack B.P., Chazin W.J., Kehl-Fie T.E., Culotta V.C. (2018). Role of Calprotectin in Withholding Zinc and Copper from Candida albicans. Infect. Immun..

[22] Li X., Hong L., Ru M., Cai R., Meng Y., Wang B., Diao H., Li L., Wu Z. (2024). S100A8/A9-activated IFNgamma(+) NK cells trigger beta-cell necroptosis in hepatitis B virus-associated liver cirrhosis. Cell. Mol. Life Sci..

[23] Huot N., Planchais C., Rosenbaum P., Contreras V., Jacquelin B., Petitdemange C., Lazzerini M., Beaumont E., Orta-Resendiz A., Rey F.A. (2023). SARS-CoV-2 viral persistence in lung alveolar macrophages is controlled by IFN-gamma and NK cells. Nat. Immunol..

[24] Xu S.W., Ilyas I., Weng J.P. (2023). Endothelial dysfunction in COVID-19: An overview of evidence, biomarkers, mechanisms and potential therapies. Acta Pharmacol. Sin..

[25] Wang Y., Shi Y., Shao Y., Lu X., Zhang H., Miao C. (2024). S100A8/A9(hi) neutrophils induce mitochondrial dysfunction and PANoptosis in endothelial cells via mitochondrial complex I deficiency during sepsis. Cell Death Dis..

[26] Ji X., Nie C., Yao Y., Ma Y., Huang H., Hao C. (2024). S100A8/9 modulates perturbation and glycolysis of macrophages in allergic asthma mice. PeerJ.

[27] Pei H., Chen J., Qu J., Lu Z. (2024). S100A9 exacerbates sepsis-induced acute lung injury via the IL17-NFkappaB-caspase-3 signaling pathway. Biochem. Biophys. Res. Commun..

[28] Moody R., Sonda S., Johnston F.H., Smith K.J., Stephens N., McPherson M., Flanagan K.L., Plebanski M. (2022). Antibodies against Spike protein correlate with broad autoantigen recognition 8 months post SARS-CoV-2 exposure, and anti-calprotectin autoantibodies associated with better clinical outcomes. Front. Immunol..

[29] Havelka A., Larsson A.O., Martensson J., Bell M., Hultstrom M., Lipcsey M., Eriksson M. (2023). Analysis of Calprotectin as an Early Marker of Infections Is Economically Advantageous in Intensive Care-Treated Patients. Biomedicines.

[30] Havelka A., Sejersen K., Venge P., Pauksens K., Larsson A. (2020). Calprotectin, a new biomarker for diagnosis of acute respiratory infections. Sci. Rep..

[31] Diehl-Wiesenecker E., Galtung N., Dickescheid J., Prpic M., Somasundaram R., Kappert K., Bauer W. (2024). Blood calprotectin as a biomarker for infection and sepsis—The prospective CASCADE trial. BMC Infect. Dis..

[32] Yokoyama Y., Ichiki T., Yamakawa T., Tsuji Y., Kuronuma K., Takahashi S., Narimatsu E., Katanuma A., Nakase H. (2024). Gut microbiota and metabolites in patients with COVID-19 are altered by the type of SARS-CoV-2 variant. Front. Microbiol..

[33] Bergmann F., Gabler C., Nussbaumer-Proll A., Wolfl-Duchek M., Blaschke A., Radtke C., Zeitlinger M., Jorda A. (2023). Early Bacterial Coinfections in Patients Admitted to the ICU With COVID-19 or Influenza: A Retrospective Cohort Study. Crit. Care Explor..

[34] Contou D., Claudinon A., Pajot O., Micaelo M., Longuet Flandre P., Dubert M., Cally R., Logre E., Fraisse M., Mentec H. (2020). Bacterial and viral co-infections in patients with severe SARS-CoV-2 pneumonia admitted to a French ICU. Ann. Intensive Care.

[35] Musuuza J.S., Watson L., Parmasad V., Putman-Buehler N., Christensen L., Safdar N. (2021). Prevalence and outcomes of co-infection and superinfection with SARS-CoV-2 and other pathogens: A systematic review and meta-analysis. PLoS ONE.

[36] Katz J., Yue S., Xue W. (2022). Herpes simplex and herpes zoster viruses in COVID-19 patients. Ir. J. Med. Sci..

[37] Giacobbe D.R., Di Bella S., Dettori S., Brucci G., Zerbato V., Pol R., Segat L., D’Agaro P., Roman-Pognuz E., Friso F. (2022). Reactivation of Herpes Simplex Virus Type 1 (HSV-1) Detected on Bronchoalveolar Lavage Fluid (BALF) Samples in Critically Ill COVID-19 Patients Undergoing Invasive Mechanical Ventilation: Preliminary Results from Two Italian Centers. Microorganisms.

[38] Garcia-Vidal C., Sanjuan G., Moreno-Garcia E., Puerta-Alcalde P., Garcia-Pouton N., Chumbita M., Fernandez-Pittol M., Pitart C., Inciarte A., Bodro M. (2021). Incidence of co-infections and superinfections in hospitalized patients with COVID-19: A retrospective cohort study. Clin. Microbiol. Infect..

[39] Bazaid A.S., Barnawi H., Qanash H., Alsaif G., Aldarhami A., Gattan H., Alharbi B., Alrashidi A., Al-Soud W.A., Moussa S. (2022). Bacterial Coinfection and Antibiotic Resistance Profiles among Hospitalised COVID-19 Patients. Microorganisms.

[40] Luke F., Orso E., Kirsten J., Poeck H., Grube M., Wolff D., Burkhardt R., Lunz D., Lubnow M., Schmidt B. (2020). Coronavirus disease 2019 induces multi-lineage, morphologic changes in peripheral blood cells. EJHaem.

[41] Mairpady Shambat S., Gomez-Mejia A., Schweizer T.A., Huemer M., Chang C.C., Acevedo C., Bergada-Pijuan J., Vulin C., Hofmaenner D.A., Scheier T.C. (2022). Hyperinflammatory environment drives dysfunctional myeloid cell effector response to bacterial challenge in COVID-19. PLoS Pathog..

[42] Silvin A., Chapuis N., Dunsmore G., Goubet A.G., Dubuisson A., Derosa L., Almire C., Henon C., Kosmider O., Droin N. (2020). Elevated Calprotectin and Abnormal Myeloid Cell Subsets Discriminate Severe from Mild COVID-19. Cell.

[43] Jonsson N., Nilsen T., Gille-Johnson P., Bell M., Martling C.R., Larsson A., Mårtensson J. (2017). Calprotectin as an early biomarker of bacterial infections in critically ill patients: An exploratory cohort assessment. Crit. Care Resusc..

[44] Parke A., Unge C., Yu D., Sunden-Cullberg J., Stralin K. (2023). Plasma calprotectin as an indicator of need of transfer to intensive care in patients with suspected sepsis at the emergency department. BMC Emerg. Med..

[45] Duggan S., Leonhardt I., Hunniger K., Kurzai O. (2015). Host response to Candida albicans bloodstream infection and sepsis. Virulence.

[46] Hetland G., Fagerhol M.K., Mirlashari M.R., Nissen-Meyer L.S.H., Croci S., Lonati P.A., Bonacini M., Salvarani C., Marvisi C., Bodio C. (2024). Elevated NET, Calprotectin, and Neopterin Levels Discriminate between Disease Activity in COVID-19, as Evidenced by Need for Hospitalization among Patients in Northern Italy. Biomedicines.

[47] Zhang H., Zhang Q., Liu K., Yuan Z., Xu X., Dong J. (2024). Elevated level of circulating calprotectin correlates with severity and high mortality in patients with COVID-19. Immun. Inflamm. Dis..

[48] Didriksson I., Lengquist M., Spangfors M., Leffler M., Sievert T., Lilja G., Frigyesi A., Friberg H., Schiopu A. (2024). Increasing plasma calprotectin (S100A8/A9) is associated with 12-month mortality and unfavourable functional outcome in critically ill COVID-19 patients. J. Intensive Care.

[49] Shokri-Afra H., Alikhani A., Moradipoodeh B., Noorbakhsh F., Fakheri H., Moradi-Sardareh H. (2021). Elevated fecal and serum calprotectin in COVID-19 are not consistent with gastrointestinal symptoms. Sci. Rep..

[50] Taylor S.P., Anderson W.E., Beam K., Taylor B., Ellerman J., Kowalkowski M.A. (2021). The Association Between Antibiotic Delay Intervals and Hospital Mortality Among Patients Treated in the Emergency Department for Suspected Sepsis. Crit. Care Med..

[51] Bone R.C. (1995). Sepsis, sepsis syndrome, and the systemic inflammatory response syndrome (SIRS). Gulliver in Laputa. JAMA.

[52] Singer M., Deutschman C.S., Seymour C.W., Shankar-Hari M., Annane D., Bauer M., Bellomo R., Bernard G.R., Chiche J.D., Coopersmith C.M. (2016). The Third International Consensus Definitions for Sepsis and Septic Shock (Sepsis-3). JAMA.

[53] https://www.covid19treatmentguidelines.nih.gov/overview/clinical-spectrum/.

[54] Evans L., Rhodes A., Alhazzani W., Antonelli M., Coopersmith C.M., French C., Machado F.R., McIntyre L., Ostermann M., Prescott H.C. (2021). Surviving Sepsis Campaign: International Guidelines for Management of Sepsis and Septic Shock 2021. Crit. Care Med..

[55] Karakike E., Giamarellos-Bourboulis E.J., Kyprianou M., Fleischmann-Struzek C., Pletz M.W., Netea M.G., Reinhart K., Kyriazopoulou E. (2021). Coronavirus Disease 2019 as Cause of Viral Sepsis: A Systematic Review and Meta-Analysis. Crit. Care Med..

[56] Garcia de Guadiana-Romualdo L., Rodriguez Rojas C., Morell-Garcia D., Andaluz-Ojeda D., Rodriguez Mulero M.D., Rodriguez-Borja E., Ballesteros-Vizoso A., Calvo M.D., Albert-Botella L., Pozo Giraldez A. (2022). Circulating levels of calprotectin, a signature of neutrophil activation in prediction of severe respiratory failure in COVID-19 patients: A multicenter, prospective study (CalCov study). Inflamm. Res..

[57] Udeh R., Advani S., de Guadiana Romualdo L.G., Dolja-Gore X. (2021). Calprotectin, an Emerging Biomarker of Interest in COVID-19: A Systematic Review and Meta-Analysis. J. Clin. Med..

[58] Toma L., Dodot M., Zgura A., Bacalbasa N., Silaghi A., Simu R., Isac T., Mercan-Stanciu A. (2022). Calprotectin in viral systemic infections-COVID-19 versus hepatitis C virus. Clin. Exp. Med..

[59] Pink I., Raupach D., Fuge J., Vonberg R.P., Hoeper M.M., Welte T., Rademacher J. (2021). C-reactive protein and procalcitonin for antimicrobial stewardship in COVID-19. Infection.

[60] Buehler P.K., Zinkernagel A.S., Hofmaenner D.A., Wendel Garcia P.D., Acevedo C.T., Gomez-Mejia A., Mairpady Shambat S., Andreoni F., Maibach M.A., Bartussek J. (2021). Bacterial pulmonary superinfections are associated with longer duration of ventilation in critically ill COVID-19 patients. Cell Rep. Med..

[61] Rakiro J., Shah J., Waweru-Siika W., Wanyoike I., Riunga F. (2021). Microbial coinfections and superinfections in critical COVID-19: A Kenyan retrospective cohort analysis. IJID Reg..

[62] Moynan D., Barkat R., Avramovic G., Morley D., O’Kelly B., Lambert J.S. (2023). The reactivation of herpesviruses in severe COVID-19; a retrospective analysis of a critical care cohort. Clin. Infect. Pract..

[63] Boers L.S., van Someren Greve F., van Hattem J.M., de Brabander J., Zwaan T., van Willigen H., Cornelissen M., de Jong M., van der Poll T., Duitman J. (2024). Pulmonary herpes simplex virus and cytomegalovirus in patients with acute respiratory distress syndrome related to COVID-19. Intensive Care Med..

[64] Willems R.J., Top J., van Santen M., Robinson D.A., Coque T.M., Baquero F., Grundmann H., Bonten M.J. (2005). Global spread of vancomycin-resistant Enterococcus faecium from distinct nosocomial genetic complex. Emerg. Infect. Dis..

[65] Raza T., Ullah S.R., Mehmood K., Andleeb S. (2018). Vancomycin resistant Enterococci: A brief review. J. Pak. Med. Assoc..

[66] Zakaria N.D., Hamzah H.H., Salih I.L., Balakrishnan V., Abdul Razak K. (2023). A Review of Detection Methods for Vancomycin-Resistant Enterococci (VRE) Genes: From Conventional Approaches to Potentially Electrochemical DNA Biosensors. Biosensors.

[67] Adriana D.N., Sugihartono T., Nusi I.A., Setiawan P.B., Purbayu H., Maimunah U., Kholili U., Widodo B., Thamrin H., Vidyani A. (2022). Role of fecal calprotectin as a hypoxic intestinal damage biomarker in COVID-19 patients. Gut Pathog..

[68] Al-Kuraishy H.M., Al-Gareeb A.I., Al-Niemi M.S., Alexiou A., Batiha G.E. (2022). Calprotectin: The Link Between Acute Lung Injury and Gastrointestinal Injury in Covid-19: Ban or Boon. Curr. Protein Pept. Sci..

[69] Tomasiuk R., Dabrowski J., Smykiewicz J., Wiacek M. (2021). Predictors of COVID-19 Hospital Treatment Outcome. Int. J. Gen. Med..

[70] Wirtz T.H., Buendgens L., Weiskirchen R., Loosen S.H., Haehnsen N., Puengel T., Abu Jhaisha S., Brozat J.F., Hohlstein P., Koek G. (2020). Association of Serum Calprotectin Concentrations with Mortality in Critically Ill and Septic Patients. Diagnostics.

[71] Idelevich E.A., Reischl U., Becker K. (2018). New Microbiological Techniques in the Diagnosis of Bloodstream Infections. Dtsch. Arztebl. Int..

