# Supplementary material for: Calprotectin, a Promising Serological Biomarker for the Early Diagnosis of Superinfections with Multidrug-Resistant Bacteria in Patients with COVID-19
Source: Int J Mol Sci. 2024 Aug 27;25(17):9294. doi: 10.3390/ijms25179294 (PMC11394900; doi:10.3390/ijms25179294)
Supplement: Supplementary file 1 [file ijms-25-09294-s001.zip › ijms-3168110-supplementary.pdf]

Table S1. Antimicrobial susceptibility of the VRE-pathogens. Data of two patients were not available because these tests were not performed in our hospital.

	VRE-Pathogen	Phenotype	Linezolid	Daptomycin
1)	*Enterococcus faecium*	Van-B	S	
2)	*Enterococcus faecium*	Van-A	S	
3)	*Enterococcus faecium*	Van-B	S	
4)	*Enterococcus faecium*	Van-B	S	
5)	*Enterococcus faecium*	Van-B	R	MIC=1 μg/ml
6)	*Enterococcus faecium*	Van-B	S	
7)	*Enterococcus faecium*	Van-A	S	
8)	*Enterococcus faecium*	Van-B	R	MIC=1 μg/ml

VRE: Vancomycin-resistant Enterococci, S: Sensitive, R: Resistant, MIC: Minimum Inhibitory Concentration
